# Both NaCl and H_2_O_2_ Long-Term Stresses Affect Basal Cytosolic Ca^2+^ Levels but Only NaCl Alters Cytosolic Ca^2+^ Signatures in *Arabidopsis*

**DOI:** 10.3389/fpls.2018.01390

**Published:** 2018-10-23

**Authors:** Lulu Liu, Zhonghao Jiang, Shu Zhang, Hongyan Zhao, Weiguang Yang, James N. Siedow, Zhen-Ming Pei

**Affiliations:** ^1^College of Life Sciences, Zhejiang University, Hangzhou, China; ^2^Center on Plant Environmental Sensing, College of Life and Environmental Sciences, Hangzhou Normal University, Hangzhou, China; ^3^Department of Biology, Duke University, Durham, NC, United States; ^4^School of Life Sciences, Zhejiang Sci-Tech University, Hangzhou, China

**Keywords:** *Arabidopsis thaliana*, calcium imaging, aequorin, calcium signaling, basal cytosolic Ca^2+^ level, salt stress, oxidative stress

## Abstract

Salinity is one of the formidable environmental factors that affect plant growth and development and constrain agricultural productivity. Experimentally imposed short-term NaCl treatment triggers a transient increase in cytosolic free Ca^2+^ concentration ([Ca^2+^]_i_) via Ca^2+^ influx across the plasma membrane. Salinity stress, as well as other stresses, induces the production of reactive oxygen species (ROS), such as H_2_O_2_. It is well established that short-term H_2_O_2_ treatment also triggers a transient increase in [Ca^2+^]_i_. However, whether and how long-term NaCl and H_2_O_2_ treatments affect the basal levels of [Ca^2+^]_i_ as well as plant responses to additional NaCl and H_2_O_2_ stresses remain poorly understood. Using an aequorin-based Ca^2+^ imaging assay, we found that the long-term treatment of *Arabidopsis* seedlings with both moderate NaCl and H_2_O_2_ in the growth media reduced the basal [Ca^2+^]_i_ levels. Interestingly, we found that the long-term treatment with NaCl, but not H_2_O_2,_ affected the responses of plants to additional NaCl stress, and remarkably the roots displayed enhanced responses while the leaves showed reduced responses. These findings suggest that plants adapt to the long-term NaCl stress, while H_2_O_2_ might be an integrator of many stresses.

## Introduction

Soil salinization impacts nearly every aspect of plant growth and development and causes enormous agricultural production losses all over the world (Hasegawa et al., 2000; Zhu, 2002, 2016). High salinity not only leads to continuing loss of arable land but also decreases crop yields. High salinity affects almost a quarter to one-third of global agricultural land, especially in irrigated areas. Previous studies show that high salinity led us to lose about 10 million hectares of agricultural land per year (Zhu, 2001; Munns and Tester, 2008; Deinlein et al., 2014). An additional challenge that compounded these losses is that agriculture needs to provide enough food for a rapidly expanding population in the world and stave off large-scale food shortages (Schroeder et al., 2013). So, it is vital to understand how plants perceive and respond to salt stress.

Researchers have conducted many studies to dissect molecular and genetic mechanisms of plants’ response to salt (NaCl) stress, in which *Arabidopsis thaliana* was widely used. Excess NaCl in plants causes hyperosmotic stress and cellular ion imbalances (Zhu, 2003; Munns and Tester, 2008). NaCl stress triggers a transient increase in cytosolic Ca^2+^ concentration ([Ca^2+^]_i_) in plants, leading to transcriptional regulation, subsequent growth, as well as developmental responses (Knight et al., 1997; Tracy et al., 2008). Although the molecular nature of initial salt stress perception is unclear, it is highly speculated that this salt-triggered rapid rise in [Ca^2+^]_i_, which lasts about 2 min, represents a sensory process in plants (Munns and Tester, 2008; Deinlein et al., 2014). This is in accordance with the notion that cytosolic Ca^2+^ is a versatile secondary messenger and a vital element in a complex signaling network responding to different abiotic and biotic stimuli, including salt stress (Knight and Knight, 2001; Ru et al., 2008; Dodd et al., 2010; Marti et al., 2013). Given that [Ca^2+^]_i_ of plants rapidly increased within a few seconds of exposure to NaCl, plant salt sensor is likely to be tightly coupled with Ca^2+^ channels.

In saline environments, salt stress also triggers other stresses such as osmotic, ionic, and oxidative stresses, and hence the growth of plants also commonly rely on their ability to cope with these stresses (Bose et al., 2015). Environmental stresses including salt stress trigger plants accumulating reactive oxygen species (ROS), and overproduction of ROS such as hydrogen peroxide (H_2_O_2_) occurs after salt stress treatment (Vaidyanathan et al., 2003; Borsani et al., 2005; Valderrama et al., 2006; Leshem et al., 2007; Miller et al., 2010; Ben Rejeb et al., 2014). As estimated from previous studies, the time constant for salt-triggered increases in [Ca^2+^]_i_ is approximately 20 s and for salt-triggered ROS is 400 s (Knight et al., 1997; Leshem et al., 2007; Jiang et al., 2013; Cao et al., 2017). It seems the rapid rise in [Ca^2+^]_i_ happens earlier than the elevation of ROS imposed by salinity. Considering ROS can also trigger increases in [Ca^2+^]_i_ (McAinsh et al., 1996; Pei et al., 2000; Leshem et al., 2007; McAinsh and Pittman, 2009; Ma and Berkowitz, 2011), it is possible that, in the signal transduction pathway of salt stress, ROS-triggered [Ca^2+^]_i_ rise perhaps functions as a feed-forward mechanism. According to recent studies of systemically propagating Ca^2+^ and ROS waves in plants, a new cell-to-cell communication pathway coupling with electric signals may help us understand how plant cells transmit long-distance signals (Gilroy et al., 2014; Kurusu et al., 2015). It should be noted that we have also found that salt-induced ROS could not trigger a detectable increase in [Ca^2+^]_i_ under the imposed experimental conditions (Jiang et al., 2013). In plants, every specific [Ca^2+^]_i_ signature is generated by the accurately manipulated activities of Ca^2+^ channels and transporters which are located in different tissues, organelles, and membranes. Cytosolic Ca^2+^ sensors include the calcineurin B-like (CBL) protein families, the Ca^2+^-dependent protein kinase (CDPK), the calmodulin (CaM), and the calmodulin-like (CML), which could monitor and decode the information loaded in [Ca^2+^]_i_ signatures, allowing plants to give a quick response and tightly adapt to the ever-changing environment (Harper and Harmon, 2005; Dodd et al., 2010; Zhu et al., 2013). Previously we analyzed that the relationship and interaction of the [Ca^2+^]_i_ increases are induced by salt stress and ROS in a short-term regime and found that NaCl-gated Ca^2+^ channels (NaC) and hydrogen peroxide (H_2_O_2_)-gated Ca^2+^ channels (HpC) are different (Jiang et al., 2013). However, whether the long-term treatment of NaCl and H_2_O_2_ on the basal [Ca^2+^]_i_ are the same with plants responses to short-term stresses remains poorly understood.

In this research, we have systematically analyzed the basal [Ca^2+^]_i_ under various long-term saline and oxidative growth conditions, and the [Ca^2+^]_i_ signatures in response to short-term stresses under these growth regimes. These findings will not only enable us to better understand how plants perceive high salt and ROS signals but also establish assays for phenotyping potential *Arabidopsis* mutants with defects in NaCl- or H_2_O_2_-induced [Ca^2+^]_i_ increases aiming to identify these sensitive Ca^2+^ channels.

## Materials and Methods

### Plant Materials and Growth Conditions

*Arabidopsis thaliana* ecotype Columbia-0 (Col-0) constitutively expressing intracellular aequorin (pMAQ2, a kind gift from Dr. M. Knight) under the control of the cauliflower mosaic virus 35S promoter was used (Knight et al., 1997; Tang et al., 2007). Seeds were sterilized with 2.5% PPM (Plant Preservative Mixture; Caisson Labs) at 4°C for 3 days. *Arabidopsis* plants were grown in 150 mm × 15 mm round Petri dishes in growth room. The culture media contained ½ Murashige and Skoog salts (MS; Sigma), 1.5% (w/v) sucrose (Sigma), and 0.8% (w/v) agar (Becton Dickinson), which was adjusted to pH 6.0 with KOH. We kept the temperature of the environmental rooms at 22 ± 2°C. The photo fluency rate of white light was ∼110 μmol m^−2^ s^−1^, and the photoperiods were 16 h light/8 h dark cycles. NaCl and H_2_O_2_ were added to the ½ MS media for saline and oxidative environments, respectively.

### Aequorin Reconstitution and Measurement of [Ca^2+^]_i_

*Arabidopsis thaliana* plants expressing cytosolic apoaequorin were used for [Ca^2+^]_i_ measurements (Knight et al., 1991; Tang et al., 2007; Yuan et al., 2014). Seedlings were grown on ½ MS medium for 9 days. Reconstitution of aequorin was conducted *in vivo* by spraying seedlings with 240 μL of 10 μM coelenterazine per Petri dish followed by incubation at 22°C in the dark for 8 h. Treatments and aequorin luminescence imaging were conducted at room temperature using a ChemiPro HT system including a cryogenically cooled and back-illuminated CCD camera or a newer version Lumazone system (Pylon1300B; Princeton Instruments) equipped with the H-800 light-tight controlled environmental box (Bio-One Scientific Instrument), liquid nitrogen auto filler (Roper Scientific or Bio-One Scientific Instrument), camera controller, and computer-equipped WinView/32 software (Roper Scientific) as described previously (Tang et al., 2007; Yuan et al., 2014). The CCD camera has a 1300 × 1340 pixel resolution and is cooled to −120°C by the cryogenic cooling system before image recording. For the basal [Ca^2+^]_i_ levels, aequorin luminescence (L) record lasted 30 min in seedlings grown under different concentrations of NaCl or H_2_O_2_. For the changes in [Ca^2+^]_i_ levels in response to NaCl and H_2_O_2_ stresses, aequorin luminescence (L) were recorded starting 50 s prior to the treatments, and luminescence images were taken every 10 s as done in our previous research (Jiang et al., 2013). In the dark, the solutions (100 mL) of H_2_O_2_ or NaCl solution was added into the Petri dish, and aequorin bioluminescence was recorded continuously (Yuan et al., 2014). Seedlings were treated with a discharging solution containing 0.9 M CaCl_2_ in 10% (v/v) ethanol and recorded for 5 min to estimate the total remaining aequorin bioluminescence (L_max_) (Knight et al., 1997; Rentel and Knight, 2004; Tang et al., 2007; Yuan et al., 2014). Finally, we used MetaMorph 7.7 to analyze the recorded image data. Considering that the relative [Ca^2+^]_i_ is relevant to the study, the [Ca^2+^]_i_ levels were shown as the ratio between aequorin luminescence (L) and total remaining aequorin (L_max_) as mentioned previously (Kiep et al., 2015; Ranf et al., 2015). All the experiments were conducted at room temperature, which was between 22 and 24°C.

### Real-Time RT-PCR

Seedlings were grown on normal ½ MS media or ½ MS media added with 40 mM NaCl or 1 mM H_2_O_2,_ for 10 days. The RNAs of leaves and roots were extracted separately, 50 mg sample of each, using RNA sample Total RNA Kit (TIANGEN). One microgram RNA was used for reverse transcription to generate the first strand of cDNA by FastKing RT Kit (TIANGEN). RT-PCR reaction was carried out using RealUniversal Color PreMix (TIANGEN). Eight-well optical PCR plate was used for each RT-PCR reaction, where each reaction contained 7.5 μL Premix, 1.5 μL forward primer, 1.5 μL reverse primer, 1.5 μL RNase-free H_2_O, and 3 μL cDNA template. The PCR cycle parameters were set as follows: 1 cycle of 15 min at 95°C, and 40 cycles of 10 s at 95°C, 20 s at 60°C, and 30 s at 72°C. *Tublin* was used as an internal control to normalize the gene expression level. Primers used in this study are listed in Supplementary Table S1. A melting curve was made after 40 cycles were completed to make sure that only single amplified products were obtained. The standard *t*-test was used for the statistical analysis.

## Results

### Long-Term Salt and Oxidative Stresses Lower the Basal [Ca^2+^]_i_

Calcium plays fundamentally essential extracellular and intracellular roles and often serves as a life and death signal in animals and plants (Berridge et al., 2003; Hofer and Brown, 2003; Dodd et al., 2010). At the resting level, the basal [Ca^2+^]_i_ is tightly maintained at a concentration that is ∼20,000-fold below the extracellular Ca^2+^ concentration (Berridge et al., 2003; Clapham, 2007; Swanson et al., 2011). Since a failure in the regulation of basal [Ca^2+^]_i_ leads to a vast array of severe diseases in humans, including cancers, neuron degeneration diseases, and cardiovascular diseases, the maintenance of adequate basal [Ca^2+^]_i_ seems to be essential for all organisms (Roderick and Cook, 2008; Sammels et al., 2010). In plants, the basal [Ca^2+^]_i_ displays diurnal oscillation patterns (Johnson et al., 1995) and is regulated by soil Ca^2+^ levels (Tang et al., 2007). In the current study, we observed significant variations of the basal [Ca^2+^]_i_ levels in these seedlings, when we used NaCl media-grown seedlings of the ecotype Col-0 expressing cytosolic apoaequorin detective in stimulus-induced [Ca^2+^]_i_ increases, similar to the osmo-sensing mutant osca1, for aequorin-based Ca^2+^ imaging analyses, we observed remarkably significant variations of the basal [Ca^2+^]_i_ levels in these seedlings. This observation trigged us to ask a question regarding whether the long-term treatments of NaCl and H_2_O_2_ affect the basal [Ca^2+^]_i_, which is in line with our previous study on the [Ca^2+^]_i_ signatures with short-term salt and ROS stresses (Jiang et al., 2013).

**FIGURE 1:**
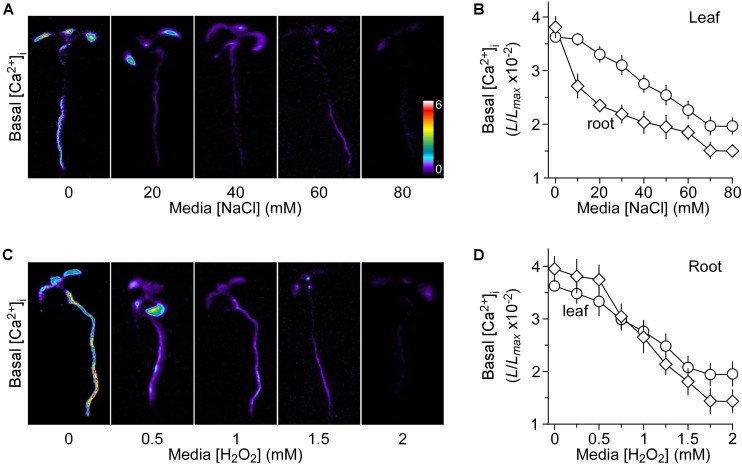
Decreases in basal [Ca^2+^]_i_ in response to long-term salt and oxidative stress. **(A,C)** Imaging of basal [Ca^2+^]_i_ in *Arabidopsis* seedlings grown under salt and oxidative stresses. Seedlings expressing aequorin were grown in the agar media containing several concentrations of NaCl (A) or H_2_O_2_ **(C)** for 9 days. Basal [Ca^2+^]_i_ in whole seedlings was analyzed by imaging aequorin luminescence (L) for 30 min. Relative [Ca^2+^]_i_ was shown as L/L_max_ (×10^−2^), and scaled by a pseudo-color bar, where L_max_ is the total aequorin luminescence. **(B,D)** Quantification of basal [Ca^2+^]_i_ in these seedlings grown under NaCl **(B)** and H_2_O_2_ **(D)** from experiments as shown in **(A,C)**, respectively. The basal [Ca^2+^]_i_ levels in leaves and roots were analyzed separately. The data from four independent experiments are shown (mean ± SEM; *n* = 20).

We grew *Arabidopsis* seedlings expressing the Ca^2+^ indicator aequorin in the MS medium containing 0 to 80 mM NaCl. It should be noted that the concentrations of NaCl were used with the consideration that the seedling growth should not be profoundly inhibited by NaCl, and thus the aequorin measurement would not be significantly affected. In other words, the seedlings looked largely normal without the apparent growth and developmental phenotypes. Aequorin bioluminescence images were taken at 30 min, and the relative value of luminescence (L/L_max_) was calculated and analyzed for the basal [Ca^2+^]_i_ (Figure 1A). Surprisingly, we observed that the basal [Ca^2+^]_i_ was decreased in response to the long-term treatment of NaCl (Figure 1A), which was in contrast to the well-known phenomenon of transient increases in [Ca^2+^]_i_ upon the short-term NaCl treatment. Plants grown on the MS medium without NaCl had a relative basal [Ca^2+^]_i_ (L/L_max_) of 3.72 ± 0.24 × 10^−2^ in the roots (Figure 1B). The basal [Ca^2+^]_i_ levels decreased under NaCl treatment in a concentration-dependent manner (Figure 1B), i.e., the higher the concentration of NaCl in the growth media the lower the basal [Ca^2+^]_i_. The basal [Ca^2+^]_i_ decreased to 1.75 ± 0.33 × 10^−2^ at 70 mM NaCl and maintained the similar value at 80 mM NaCl in the roots. We also noted that the reduction of basal [Ca^2+^]_i_ in the roots was more than that in the leaves (Figure 1B). To figure out whether and how the changes in the basal [Ca^2+^]_i_ affect NaCl- or H_2_O_2_-triggered transient increases in [Ca^2+^]_i_, we should identify an optimum concentration of NaCl in the growth media, as described previously, for potential up- and down-regulation. The ideal concentration should be used to produce about half of the maximum level of the basal [Ca^2+^]_i_ (Jiang et al., 2013). Figure 1B shows that the concentration of NaCl required for a half-maximal response was ∼40 mM, and hence we chose 40 mM NaCl for the subsequent experiments.

Besides, we researched the basal [Ca^2+^]_i_ levels in response to added H_2_O_2_ in the growth media. Seedlings were grown in MS medium with different concentrations of H_2_O_2_, which was from 0 to 2 mM. We also analyzed the basal [Ca^2+^]_i_ as described above for NaCl. Interestingly, the long-term treatment of H_2_O_2_ also lowered the basal [Ca^2+^]_i_ levels in a dose-dependent manner (Figures 1C,D). Similarly, the basal [Ca^2+^]_i_ levels were more sensitive to H_2_O_2_ in the roots than in the leaves (Figure 1D). Finally, 1 mM H_2_O_2_ was chosen as the optimum concentration, in which the magnitude of [Ca^2+^]_i_ was similar to that in the media with 40 mM NaCl.

### Response on [Ca^2+^]_i_ Induced by Short-Term Stimulus After Long-Term NaCl and H_2_O_2_ Pretreatment

**FIGURE 2:**
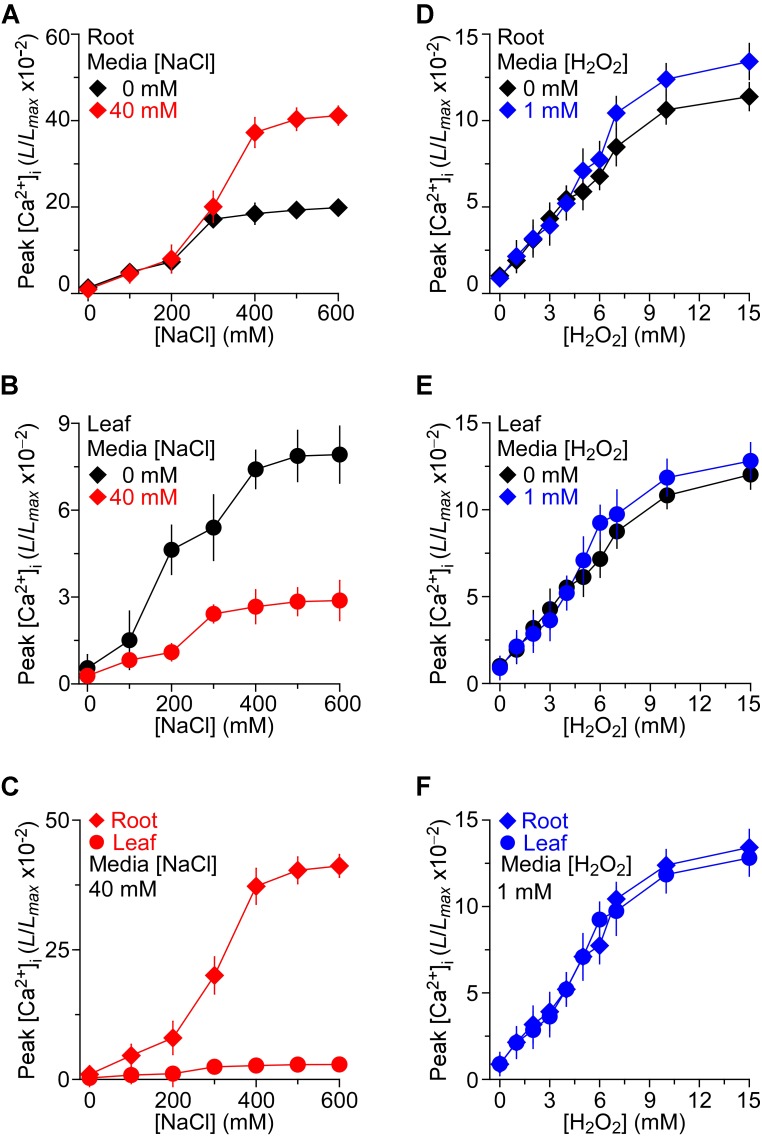
The effects of NaCl and H_2_O_2_ growth environments on increases in [Ca^2+^]_i_ induced by NaCl and H_2_O_2_, respectively. **(A–C)** *Arabidopsis* seedlings expressing aequorin grown under 0 or 40 mM NaCl for 9 days were treated with several concentrations of NaCl, and aequorin luminescence images were taken every 10 s, for 200 s. Data are averaged peak values of increases in [Ca^2+^]_i_ induced by NaCl in roots **(A)** and leaves **(B)**, respectively. The sensitivities of [Ca^2+^]_i_ to NaCl in leaves and roots from seedlings grown under 40 mM NaCl are directly compared in **(C)**. Data from four independent experiments are shown (mean ± SEM; *n* = 20). **(D–F)** Seedlings grown under 0 or 1 mM H_2_O_2_ for 9 days were treated with several concentrations of H_2_O_2_, and aequorin images were taken every 10 s, for 200 s. Data are averaged peak values of [Ca^2+^]_i_ increases in roots **(D)** and leaves **(E)**, respectively. The sensitivities of [Ca^2+^]_i_ to H_2_O_2_ in leaves and roots grown under 1 mM H_2_O_2_ are directly compared in **(F)**. Data from four independent experiments are shown (mean ± SEM; *n* = 20).

We then asked whether the long-term treatment of *Arabidopsis* with NaCl affects plants’ response to short-term NaCl stress. In other words, we asked whether plants are sensitized or desensitized to NaCl stress when they grow in NaCl media. To determine NaCl-induced [Ca^2+^]_i_ increases in *Arabidopsis* seedling grown under salt environments and to investigate these [Ca^2+^]_i_ increases in roots and leaves separately, we detected the temporal dynamics of NaCl-induced [Ca^2+^]_i_ increases without NaCl in the grown media as controls for further comparison. After the application of NaCl, the [Ca^2+^]_i_ elevated immediately, reached a peak at about 20 s, and then declined gradually as described previously (Jiang et al., 2013). Several concentrations of NaCl, ranging from 0 to 600 mM, were applied and the peak values of [Ca^2+^]_i_ were plotted as a function of the applied NaCl concentrations in the roots (Figure 2A) and leaves (Figure 2B), separately, for celerity. The magnitudes of [Ca^2+^]_i_ were increased with increases in the concentration of NaCl (Figures 2A,B). The magnitudes of NaCl-induced [Ca^2+^]_i_ increases were higher in the roots than in the leaves (Figure 2C), similar to those seen in a previous study (Tracy et al., 2008), while similar to another previous finding (Jiang et al., 2013), we found that half of the maximum amplitude [Ca^2+^]_i_ response was about 200 mM, both in the roots and leaves.

To access how the consistent presence of NaCl stress in the growth media affects the plants’ response to additional NaCl stress, the seedlings that were grown in the 40 mM NaCl media were treated with gradient concentrations of NaCl from 0 to 600 mM. We found that the [Ca^2+^]_i_ increases in response to NaCl in MS medium containing 40 mM NaCl were qualitatively consistent with the response to NaCl in general MS, and a higher NaCl induced a greater peak of [Ca^2+^]_i_ (Figures 2A,B). When changes in [Ca^2+^]_i_ were detectable in the leaf which was grown in MS containing 40 mM NaCl, the increasing trend was smaller compared with those in MS. On the contrary, the changes in [Ca^2+^]_i_ that were detectable in the root which grew in salt environment were higher. The root was more sensitive than the leaf under the salt environment compared with the seedling grown in MS (Figure 2C).

Besides, to investigate thoroughly the relationship of H_2_O_2_-induced [Ca^2+^]_i_ increases in the root and leaf, the temporal dynamics of [Ca^2+^]_i_ increases triggered by H_2_O_2_ were further compared. Seedlings were stimulated by concentrations of H_2_O_2_ from 0 to 15 mM in MS. After the application of H_2_O_2_, the [Ca^2+^]_i_ increased immediately and reached a peak at about 50 s, and then declined gradually. As expected, H_2_O_2_ induced increases in [Ca^2+^]_i_ in a dose-dependent manner (Figures 2D,E). Current results were similar to those reported previously (Jiang et al., 2013). We found that [Ca^2+^]_i_ increased in response to H_2_O_2_ and the magnitudes of [Ca^2+^]_i_ depended on the concentration of H_2_O_2_, the higher the concentration of H_2_O_2_ the higher the magnitude of [Ca^2+^]_i_ (Figures 2D,E). However, after the application of H_2_O_2_, the [Ca^2+^]_i_ increased immediately, and [Ca^2+^]_i_ increase in the root was similar to that in the leaf, which was different from the NaCl -induced [Ca^2+^]_i_ increases (Figures 2D,E). To further study how the changes in H_2_O_2_-induced [Ca^2+^]_i_ increases in the oxidative environment, the seedlings were grown in MS medium containing 1 mM H_2_O_2_. We then found that the increases in [Ca^2+^]_i_ in response to H_2_O_2_ from 0 to 15 mM in MS medium containing 1 mM H_2_O_2_ were qualitatively the same as those grown in the control MS media. The difference of [Ca^2+^]_i_ between the root and leaf in the oxidative environment was a little higher than that in the general environment (Figures 2D,E). Additionally, the [Ca^2+^]_i_ increases induced by H_2_O_2_ (0–15 mM) in root were similar to these in leaf of seedlings grown in the oxidative environment (Figure 2F).

### Long-Term NaCl Treatment Alters Short-Term Response of [Ca^2+^]_i_ to NaCl but Not H_2_O_2_

**FIGURE 3:**
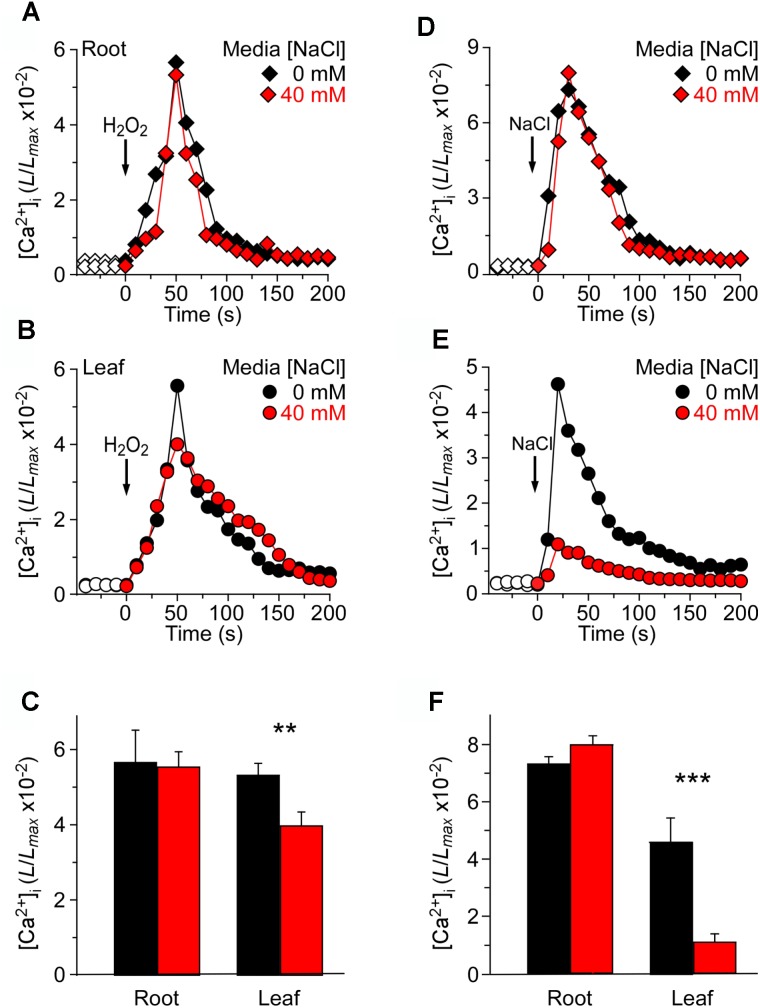
Long-term treatment of seedlings by NaCl in the growth media affects [Ca^2+^]_i_ increases induced by NaCl but not H_2_O_2_. **(A–C)** *Arabidopsis* seedlings grown in MS media containing 0 or 40 mM NaCl for 9 days were subjected to a 4 mM H_2_O_2_ treatment at 0 s, and aequorin luminescence images in roots **(A,C)** and leaves **(B,C)** were taken every 10 s throughout the treatment. Representative recordings from individual seedlings were shown. Similar results were seen in four independent experiments using 20 seedlings. **(D–F)** Similar seedlings as in **(A,B)** were treated with 200 mM NaCl at time zero, and aequorin images in roots **(D,F)** and leaves **(E,F)** were recorded continuously throughout the treatment. Representative recordings from individual seedlings were shown. Similar results were seen in four independent experiments using 20 seedlings (mean ± SD; *n* = 20; ^∗∗∗^*P* < 0.001; ^∗∗^0.001 < *P* < 0.01).

Previous studies have reported that the NaCl concentration for a half-maximal response was about 200 mM, and the magnitude of [Ca^2+^]_i_ was similar to that induced by 4 mM H_2_O_2_ (Jiang et al., 2013). The seedlings grown in MS as a control and responses to the stimulus measured using a ChemiPro system were also presented. To further study the changes in [Ca^2+^]_i_ induced by NaCl and H_2_O_2_ in salt environment, we applied 200 mM NaCl or 4 mM H_2_O_2_ to stimulate seedlings grown in MS medium and MS medium containing 40 mM NaCl, respectively (Figure 3). The [Ca^2+^]_i_ increased immediately and reached the peak at approximately 50 s, and then gradually declined. In the roots, the peak [Ca^2+^]_i_ reached 5.64 ± 0.31 (×10^−2^) in MS medium, which was similar to the peak [Ca^2+^]_i_ in seedlings grown in salt medium [5.31 ± 0.20 (×10^−2^)] (Figure 3A). In the leaves, the peak value reached 5.52 ± 0.22 (×10^−2^) in MS medium, which was similar to the root (Figure 3B). Nevertheless, the [Ca^2+^]_i_ increases were markedly declined in the leaf of the seedlings grown in salt medium (Figure 3C).

At the same time, we analyzed increases in [Ca^2+^]_i_ in response to 200 mM NaCl in seedlings grown in MS medium and MS medium containing 40 mM NaCl. After the application of 200 mM NaCl, [Ca^2+^]_i_ was increased immediately, reached a peak at about 20 s, and then declined gradually (Figures 3D,E). Interestingly, although in the root the peak [Ca^2+^]_i_ reached 7.32 ± 0.35 (×10^−2^) in the MS medium control, which was similar to the peak [Ca^2+^]_i_ in the salt medium condition [7.99 ± 0.32 (×10^−2^)] (Figure 3D), the peak [Ca^2+^]_i_ was decreased significantly in the leaf under the salt medium condition compared with MS medium (Figures 3E,F). Also, we found that NaCl-induced [Ca^2+^]_i_ increases in root reached 4.63 ± 0.19 (×10^−2^), which was higher than that in leaf [1.09 ± 0.11 (×10^−2^)], although the [Ca^2+^]_i_ increases of whole seedlings were similar to those under H_2_O_2_ treatment.

### Long-Term H_2_O_2_ Treatment Does Not Alter H_2_O_2_- or NaCl-Induced [Ca^2+^]_i_ Increases

**FIGURE 4:**
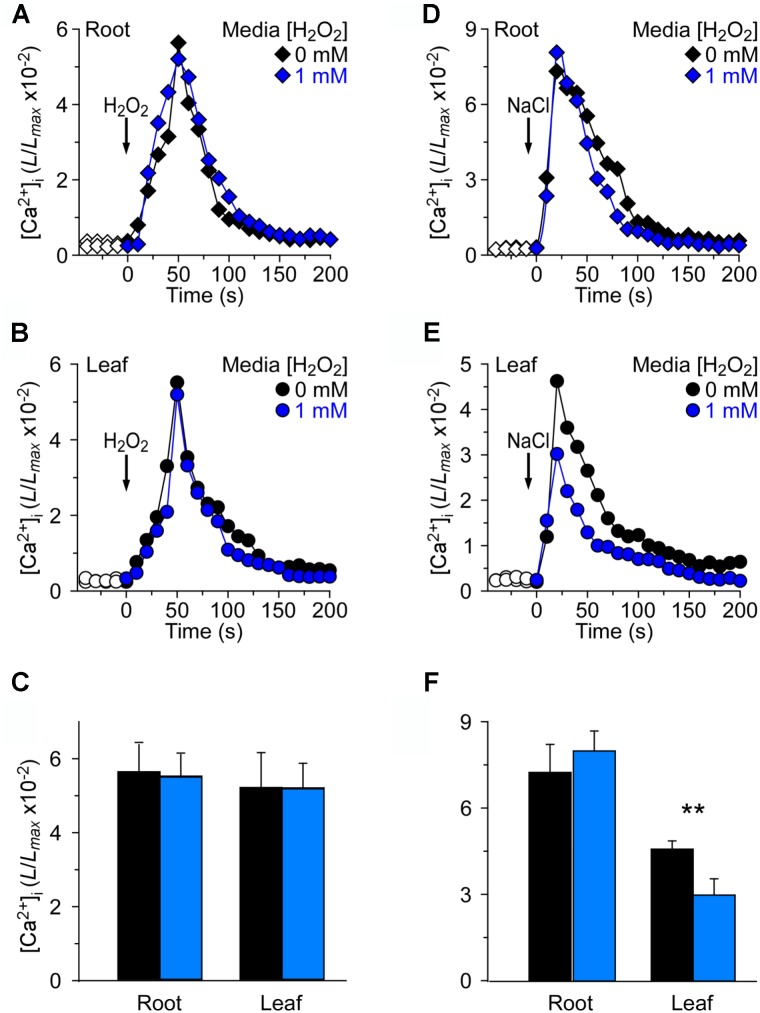
Long-term treatment of plants by H_2_O_2_ in the growth media has negligible effects on [Ca^2+^]_i_ increases induced by either H_2_O_2_ or NaCl. **(A–C)** *Arabidopsis* seedlings grown in MS media containing 0 or 1 mM H_2_O_2_ for 9 days were subjected to a 4 mM H_2_O_2_ treatment at 0 s, and aequorin luminescence images in roots **(A,C)** and leaves **(B,C)** were taken every 10 s throughout the treatment. Representative recordings from individual seedlings were shown. Similar results were seen in four independent experiments using 20 seedlings. **(D–F)** Similar seedlings as in **(A,B)** were treated with 200 mM NaCl at time zero, and aequorin images in roots **(D,F)** and leaves **(E,F)** were recorded continuously throughout the treatment. Representative recordings from individual seedlings were shown. Similar results were seen in four independent experiments using 20 seedlings (mean ± SD; *n* = 20; ^∗∗^0.001 < *P* < 0.01).

We wished to investigate further how the changes in [Ca^2+^]_i_ were triggered by NaCl and H_2_O_2_ in the oxidative environment. For this, 4 mM H_2_O_2_ and 200 mM NaCl were applied to seedlings grown in MS medium and MS medium with 1 mM H_2_O_2_, respectively (Figure 4). By analogy, we found that in the root, 4 mM H_2_O_2_ induced an immediate and rapid rise in the [Ca^2+^]_i_ which resulted in similar peaks at about 50 s under MS medium and H_2_O_2_ medium conditions, and then declined gradually (Figure 4A). We also found that the temporal dynamics of [Ca^2+^]_i_ increases was similar in both the root and the leaf (Figures 4A–C). Equally we analyzed the changes in NaCl-induced [Ca^2+^]_i_ increases in the oxidative environment. The [Ca^2+^]_i_ in response to 200 mM NaCl under MS medium and MS medium containing 1 mM H_2_O_2_ was examined. The [Ca^2+^]_i_ increased immediately in the root after being treated with 200 mM NaCl, and reached a peak of 7.32 ± 0.33 (×10^−2^) in normal medium and 8.07 ± 0.26 (×10^−2^) in H_2_O_2_ medium (Figure 4D). However, the [Ca^2+^]_i_ increases in the leaf were lower than that in the root. Additionally, after 200 mM NaCl treatment, the peak [Ca^2+^]_i_ that was increased in the H_2_O_2_ medium had a remarkable decline compared to that in MS medium (Figure 4F).

To further assess the reason why the root is more sensitive to NaCl but not H_2_O_2_, we measured the expression patterns of Ca^2+^ signal relative marker genes, such as SOS1, SOS2, SOS3, TPC1, ZAT12, and RBOHD in both the leaf and the root. For ROS stress marker genes, ZAT12, RBOHD, and TPC1, the ZAT12 and RBOHD were all up-regulated both in the leaf and the root, but the TPC1 relative expression was down-regulated in both the leaf and the root (Supplementary Figures S1A,B). It is known that TPC1 expelled Ca^2+^ from the vacuole to the cytosol in *Arabidopsis* thaliana, which contributed to long-distance Ca^2+^ signature (Evans et al., 2016; Guo et al., 2017). The expression of TPC1 was down-regulated, thus Ca^2+^ released from the vacuole might be also decreased. This might lead to higher cytosolic basal Ca^2+^ levels, which is clearly not one of the reasons for lower cytosolic Ca^2+^ seen in Figure 1. Meanwhile, during long-term salt stress, the marker genes, such as SOS1, SOS2, and SOS3, were all up-regulated much more in the root compared to that in the leaf (Supplementary Figures S1C,D). For marker gene TPC1, the expression in the root was reduced, while it was increased in the leaf (Supplementary Figures S1C,D). The TPC1 expression levels may be associated with Ca^2+^ signaling rather than the basal Ca^2+^ levels, and more so in the leaf with respect to salt stress (Supplementary Figure S1).

## Discussion

Calcium is a critical second messenger in signal transduction in animals and plants (Berridge et al., 2003; Clapham, 2007; McAinsh and Pittman, 2009; Ward et al., 2009). Previous studies have shown that specific stimuli can trigger unique temporal and spatial patterns of [Ca^2+^]_i_, known as “[Ca^2+^]_i_ signatures,” which are thought to function in most aspects of plant growth and development (Tang et al., 2007; Dodd et al., 2010; Jiang et al., 2013). Cytosolic Ca^2+^ concentrations can be monitored by imaging the Ca^2+^ indicator aequorin, and the intensity of bioluminescence only depends on the Ca^2+^ concentration. It is well-known that the basal [Ca^2+^]_i_ is maintained at 100 nM and about 20,000-fold below the extracellular Ca^2+^ concentration (Epstein, 1961; Berridge et al., 2003; Hepler, 2005; Clapham, 2007; Swanson et al., 2011). Environmental stimuli activate the Ca^2+^ channels in general located at the plasma membrane and/or endomembranes, leading to the increases in [Ca^2+^]_i_ (Hetherington and Brownlee, 2004; Ward et al., 2009). The Ca^2+^ signature encodes information from the environmental stimuli and is then decoded by intracellular Ca^2+^ sensors, such as CBL proteins and calmodulins, which initiate the downstream events (Clapham, 2007; Zhu et al., 2013). Over the past decades, a significant progress has been made in understanding the changes in [Ca^2+^]_i_ that were induced by various abiotic and biotic stresses in plants, including the responses to salt stress, drought, oxidative stress, high and low temperatures, mechanical wounding, and pathogen elicitors (McAinsh and Pittman, 2009; Dodd et al., 2010; Ma and Berkowitz, 2011; Kaya et al., 2014; Yuan et al., 2014). In line with this, we have isolated a mutant with defects in osmotic stress-induced [Ca^2+^]_i_ increases and identified the osmosensor OSCA1 (Yuan et al., 2014). Interestingly, many abiotic and biotic stresses also induce the accumulation of ROS and generate oxidative stresses, which play an important role in many aspects of plant growth and development (Wise and Naylor, 1987; Mittler, 2002; Vranova et al., 2002; Suzuki et al., 2012; Kaya et al., 2014). For instance, it has been known that salt stress enhances the accumulation of ROS in plants, and hydrogen peroxide has been proposed to function in salt-triggered calcium wave propagations (Vaidyanathan et al., 2003; Borsani et al., 2005; Valderrama et al., 2006; Leshem et al., 2007; Miller et al., 2010; Choi et al., 2016).

It has long been hypothesized that salt stress-triggered increases in [Ca^2+^]_i_ was involved in the perception of the salt signal, even though much remains to be discovered about the properties of the salt-activated Ca^2+^ permeable channel and its molecular nature (Zhu, 2001; Munns and Tester, 2008). Additionally, the molecular mechanisms of ROS sensing in plant cells are still unknown, and probably ROS activation of Ca^2+^ permeable channels may serve as an ROS perception process (Pei et al., 2000; Waring, 2005; Miller et al., 2010). Note that the rapid rise in NaCl-induced [Ca^2+^]_i_ as well as the NaCl-triggered overproduction of ROS possibly function as critical integrators, mediating stress signal perception, signal transduction, and adaptation (Xie et al., 2011; Ma et al., 2012; Jiang et al., 2013; Ben et al., 2015; Köster et al., 2018). The salt stress-induced [Ca^2+^]_i_ increases precede the production of H_2_O_2_ signaling molecule (Yang and Poovaiah, 2002). NaCl and H_2_O_2_ induce [Ca^2+^]_i_ increases and activate distinct Ca^2+^ permeable channels (Jiang et al., 2013), suggesting that calcium plays a central role in NaCl and H_2_O_2_ signal transduction pathways in plants.

Here we found that basal [Ca^2+^]_i_ decreased under salt and oxidative stress environments (Figure 1). Considering that the basal [Ca^2+^]_i_ levels are maintained at approximately 100 nM, the low basal [Ca^2+^]_i_ levels in plants grown in NaCl medium and H_2_O_2_ medium, seen in this study (Figure 1), indicates that the low levels may allow plants to adapt well to salt and oxidative environmental stresses. The Ca^2+^ sensor proteins with diverse Ca^2+^ affinities, subcellular localizations, and downstream target specificities perceive [Ca^2+^]_i_ changes and transduce them into downstream signaling responses (Hashimoto and Kudla, 2011). It seems essential for plants to maintain a lower basal [Ca^2+^]_i_ under stressed environmental conditions. It is possible that the Ca^2+^ sensors allow the plant to tightly control the appropriate adaptation to changes and provide a robust response to additional stimuli from the environment. The lower basal [Ca^2+^]_i_ can only activate high affinity Ca^2+^ sensors, and thus proportionally promote the high-affinity-Ca^2+^-sensor-mediated signaling pathways. It remains to be addressed why the basal [Ca^2+^]_i_ of the root is lowered more than that of the leaf in NaCl medium but the [Ca^2+^]_i_ of the root and leaf is similar in H_2_O_2_ medium (Figures 1B,D). Roots grow in the salt stress soil environment, but the leaf is almost not directly contacted with the salt in the soil. Based on the previous research, the salt is absorbed from the site of application of the gel, which triggers the propagation of the Ca^2+^ wave and induction of systemic molecular responses in the leaf that are unlikely to reflect direct responses to salt stress (Choi et al., 2014). We consider the initial Ca^2+^ signatures as the main springboard to further research.

Through the application of different concentrations of stimuli to plants, the relationship between the strength of stimulus and magnitude of [Ca^2+^]_i_ could be found. The magnitudes of [Ca^2+^]_i_ increases rely on the strength of stimulus, wherein, higher the strength applied the more considerable is the rise in [Ca^2+^]_i_, which is observed until the saturation point is reached (Figure 2), and this is consistent with previous reports (Tracy et al., 2008; Jiang et al., 2013). Therefore, the correlation between stimulus strengths and [Ca^2+^]_i_ magnitudes may be a common dose effect in plants. Analysis of the dose effect of NaCl-induced [Ca^2+^]_i_ increases demonstrated that the [Ca^2+^]_i_ in response to NaCl in the root is dramatically stronger than that in the leaf, and the [Ca^2+^]_i_ magnitudes between root and leaf are further elevated when the seedlings are grown in the NaCl medium (Figures 2A–C), indicating a sensitization mechanism under the imposed experimental conditions. In contrast, in the presence and absence of H_2_O_2_ in the medium, no differences of the [Ca^2+^]_i_ were found between the roots and leaves in the dose effects of H_2_O_2_ in both normal environmental and oxidative conditions, which means that the root and the leaf are neither sensitized nor desensitized when the seedlings were grown in oxidative medium (Figures 2D–F).

We have investigated the spatiotemporal patterns of the NaCl- and H_2_O_2_-induced [Ca^2+^]_i_ increases and demonstrated that both NaCl and H_2_O_2_ evoke a transient increase in [Ca^2+^]_i_ which then decays to a new resting level (Figures 3, 4), similar to those seen in previous studies (Knight et al., 1997; Rentel and Knight, 2004; Jiang et al., 2013). The [Ca^2+^]_i_ elevations in roots from seedlings grown under 40 mM NaCl medium were similar to those from control medium-grown seedlings after application of H_2_O_2_ and NaCl stimulus, respectively (Figure 3). However, the [Ca^2+^]_i_ elevations within the leaf, induced by both H_2_O_2_ and NaCl, significantly declined in NaCl medium compared to control medium. Moreover, the [Ca^2+^]_i_ elevations of leaf stimulated by NaCl reduced more than that stimulated by H_2_O_2_ (Figures 3C,F). These results indicate that the sensitivity of leaf is reduced in salt environmental stress. It is most likely that the NaCl-evoked Ca^2+^ signature within the leaf might be adapted to salt environmental conditions, but the H_2_O_2_-evoked Ca^2+^ signature within the leaf might be only partly adapted to salt environmental conditions. Furthermore, after application of H_2_O_2_ and NaCl, respectively, the [Ca^2+^]_i_ elevations of roots in the H_2_O_2_ medium are similar to those in NaCl medium (Figure 4). However, the H_2_O_2_-induced [Ca^2+^]_i_ in leaves was similar to that in roots (Figures 4A,C) and the NaCl-induced [Ca^2+^]_i_ in leaves has a marked decline in the H_2_O_2_ medium (Figure 4F). These results indicate that the NaCl-evoked Ca^2+^ signatures within the leaf might partly adapt to oxidative environmental stress condition with significant desensitization, while both the root and the leaf that are stimulated by H_2_O_2_ under the oxidative environmental conditions can maintain the same sensitivity as in normal environmental conditions.

Our results suggest that the root is the primary sensing tissue to perceive salt stress, and is more sensitive to salt stress when the seedlings are grown in the salt environment. Previous studies have shown that plants have stronger reactions on pre-exposure to drought stress to reduce water loss from leaves (Ding et al., 2012). In addition, plants have long-term somatic memory in epigenome (Sani et al., 2013). With respect to [Ca^2+^]_i_ signaling, plants exhibit rapid hyperosmotic-induced Ca^2+^ responses on pre-exposure to osmotic stress, also called “osmo-sensory potentiation” (Stephan et al., 2016). We found similar potentiation phenomenon in salt stress in the root but desensitization in the leaf. However, the oxidative stresses are different from salt and osmotic stresses – no potentiation and desensitization were observed. It is possible that the mechanistic aspects of salt stress (ionic and osmotic stresses) are shared with osmotic stress. When plants suffer from salt stress, they have to maintain enough [Ca^2+^]_i_ to transduce the Ca^2+^ signatures to trigger the downstream events in the whole plants, but too much [Ca^2+^]_i_ can also become toxic. So we speculate that in the process of plants to adapt to salt stress, the basal [Ca^2+^]_i_ decreases with pre-exposure to salt stress environment, leading to weaker Ca^2+^ signaling pathways. We have known that salt stress can trigger the SAA (Systemic acquired acclimation) (Gilroy et al., 2014), which enable whole plants to prepare all of their tissues and cells to an upcoming challenge, even though that may initially only be sensed by root tissues. It may indicate why the basal [Ca^2+^]_i_ in the leaf are decreased for the future upcoming stimulates.

In comparison with plants that cope with salt stress, our data showed that in the process of plant adaption to ROS stress, they have a delicate ROS balance between their production and scavenging, in which ROS does not need to be stored in a particular compartment and could be rapidly generated and/or removed anywhere in the cell or the apoplast (Choi et al., 2014). We have established that the magnitude of salt stress-induced [Ca^2+^]_i_ increases and the ROS-induced [Ca^2+^]_i_ increases are affected by both salt and oxidative environments. We also studied the relationship between salt- and ROS-induced [Ca^2+^]_i_ increases in leaves and roots, respectively. Our data suggest that previous studies on NaCl- and H_2_O_2_-induced [Ca^2+^]_i_ increases are much more complicated in leaf and root under the salt and oxidative environments (Knight et al., 1997; Rentel and Knight, 2004; Jiang et al., 2013). By imaging [Ca^2+^]_i_ changes, we found the basal [Ca^2+^]_i_ decrease was dependent on the saline and oxidative environments. Detailed image analyses and comparisons of the [Ca^2+^]_i_ increases suggest that the roots are more sensitive than the leaves in response to environmental stress conditions. The increases evoked by NaCl are different from those by H_2_O_2_ in salt and oxidative environmental stresses, respectively, indicating that there is a different mechanism in the plant evolution process in response to both long-term and transient stress.

## Author Contributions

ZJ and Z-MP designed the experiments. LL performed the research. LL, ZJ, SZ, HZ, and WY collected and analyzed the data. LL, ZJ, JS, and Z-MP wrote the manuscript. All authors discussed the results and contributed to the manuscript.

## Conflict of Interest Statement

The authors declare that the research was conducted in the absence of any commercial or financial relationships that could be construed as a potential conflict of interest.

## Supplementary Material

FIGURE S1 Long-term H_2_O_2_ and NaCl treatment alters relative genes expression. **(A,B)** *Arabidopsis* seedlings grown in MS media containing 0 or 1 mM H_2_O_2_ and 40 mM NaCl for 10 days. We measured several salt stress relative gene markers such as SOS1, SOS2, SOS3, and TPC1 in leaf **(A)** and root **(B)**. They are all up-regulated in root except TPC1, and they are all up-regulated in leaf. Similar results were seen in four independent experiments using 40 seedlings. **(C,D)** Similarly, we measured ROS stress relative gene markers such as ZAT12, RBOHD, and TPC1 in root and leaf. The ZAT12 and RBOHD are all up-regulated both in leaf **(C)** and root **(D)**, but the TPC1 relative expression is down-regulated both in leaf **(C)** and root **(D)**. Similar results were seen in four independent experiments using 40 seedlings.

TABLE S1 Marker Gene Primer.

## Abbreviations

AQ: aequorin
[Ca^2+^]_i_: cytosolic free Ca^2+^ concentration
CaM: calmodulin
CBL: calcineurin B-like
CDPK: Ca^2+^-dependent protein kinase
CML: calmodulin-like
HpC: hydrogen peroxide (H_2_O_2_)-gated Ca^2+^ channels
NaC: NaCl-gated Ca^2+^ channels
ROS: reactive oxygen species
