# Supplementary figures and images for: Both NaCl and H2O2 Long-Term Stresses Affect Basal Cytosolic Ca2+ Levels but Only NaCl Alters Cytosolic Ca2+ Signatures in Arabidopsis
Source: Front Plant Sci. 2018 Oct 23;9:1390. doi: 10.3389/fpls.2018.01390 (PMC6206402; doi:10.3389/fpls.2018.01390)

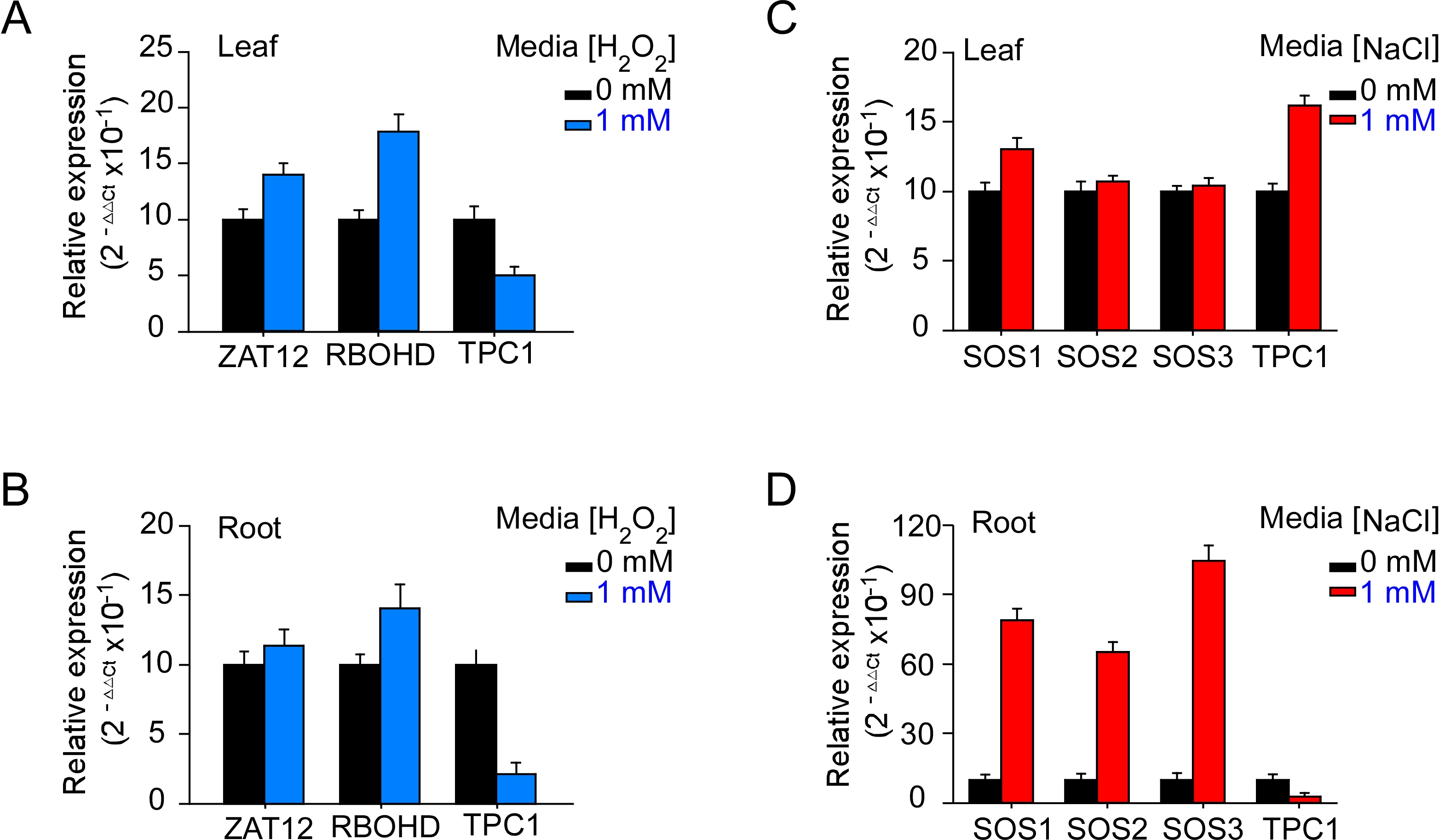

Supplement: FIGURE S1 — Long-term H2O2 and NaCl treatment alters relative genes expression. (A,B) Arabidopsis seedlings grown in MS media containing 0 or 1 mM H2O2 and 40 mM NaCl for 10 days. We measured several salt stress relative gene markers such as SOS1, SOS2, SOS3, and TPC1 in leaf (A) and root (B). They are all up-regulated in root except TPC1, and they are all up-regulated in leaf. Similar results were seen in four independent experiments using 40 seedlings. (C,D) Similarly, we measured ROS stress relative gene markers such as ZAT12, RBOHD, and TPC1 in root and leaf. The ZAT12 and RBOHD are all up-regulated both in leaf (C) and root (D), but the TPC1 relative expression is down-regulated both in leaf (C) and root (D). Similar results were seen in four independent experiments using 40 seedlings. [file Image_1.JPEG]
