# Supplementary material for: Both NaCl and H2O2 Long-Term Stresses Affect Basal Cytosolic Ca2+ Levels but Only NaCl Alters Cytosolic Ca2+ Signatures in Arabidopsis
Source: Front Plant Sci. 2018 Oct 23;9:1390. doi: 10.3389/fpls.2018.01390 (PMC6206402; doi:10.3389/fpls.2018.01390)
Supplement: TABLE S1 — Marker Gene Primer. [file Table_1.DOCX]

| Marker Gene | Forward (5’-3’) | Reverse (5’-3’) |
| --- | --- | --- |
| UBQ10 | CACACTCCACTTGGTCTTGCGT | TGGTCTTTCCGGTGAGAGTCTTCA |
| SOS1 | CTTCTTCCTCTGTGTTGTTGC | GAAGACGAATCGGTCGCTT |
| SOS2 | AAGCTATGTTCGAAACTGGAAAAC | TGGATTTAAGTTGGGATCAAAACG |
| SOS3 | AGAAGGGTGTGTTTGTATGGG | GAAGCTCGGGATCCTCATATC |
| TPC1 | GCTCTATTGGCGTACAGGTCTTTG | GAAGAGTGTGACCATTCCATTGG |
| ZAT12 | AACACAAACCACAAGAGGATCA | AAGCATCAAACAATTCGCCG |
| RBOHD | CCTATGAGCCGATGGAAAAA | ACAATGCCAGTCCATCCTTC |

**Table1 Marker Gene Primer**
